# 2,2′-Dihydroxy-3,3′-[(1*E*,1′*E*)-hydrazine-1,2-diylidenedimethylidyne]dibenzoic acid *N*,*N*-dimethylformamide disolvate

**DOI:** 10.1107/S1600536808043134

**Published:** 2008-12-24

**Authors:** Sheng-Sen Zhang, Geng-Jin-Sheng Cheng, Yu Lei, Yin-Bao Li

**Affiliations:** a College of Pharmacy, Gannan Medical University, Ganzhou, Jiangxi 341000, People’s Republic of China

## Abstract

The title compound, C_16_H_12_N_2_O_6_·2C_3_H_7_NO, lies across a crystallographic inversion centre which is situated at the midpoint of the central N—N bond. The substitution at the C=N bond adopts a *trans* configuration and it is essentially coplanar with the benzene ring [N—C—C—C torsion angles = −173.9 (4) and 6.4 (6)°]. All torsion angles involving non-H atoms are close to 180°. Intra­molecular O—H⋯O and weak C—H⋯O hydrogen bonds form *S*(6) and *S*(5) ring motifs, respectively, while inter­molecular O—H⋯O and weak C—H⋯O hydrogen bonds connect the Schiff base mol­ecule to solvent dimethyl­formamide mol­ecules.

## Related literature

For information on Schiff base ligands, their complexes and their applications, see, for example: Pal *et al.* (2005 ▶); Hou *et al.* (2001 ▶); Ren *et al.* (2002 ▶). For bond-length data, see: Allen *et al.* (1987 ▶). For the structures and properties of related azine organic and metallorganic compounds, see, for example: Dreuw *et al.* (2005 ▶); Chattopadhyay *et al.* (2008 ▶); Cucos *et al.* (2006 ▶); Fu (2007 ▶); Mijanuddina *et al.* (2004 ▶); Sreerama *et al.* (2007 ▶); Butcher *et al.* (2007 ▶). For hydrogen-bond motifs, see: Bernstein *et al.* (1995 ▶).
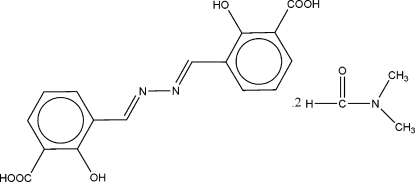


## Experimental

### 

#### Crystal data

C_16_H_12_N_2_O_6_·2C_3_H_7_NO
*M* *_r_* = 474.47
Monoclinic, 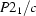

*a* = 5.9136 (12) Å
*b* = 10.837 (2) Å
*c* = 18.991 (4) Å
β = 98.96 (3)°
*V* = 1202.2 (4) Å^3^
*Z* = 2
Mo *K*α radiation
μ = 0.10 mm^−1^
*T* = 295 K
0.36 × 0.20 × 0.16 mm

#### Data collection

Bruker SMART CCD area-detector diffractometer
Absorption correction: multi-scan (*SADABS*; Sheldrick, 1996 ▶) *T*_min_ = 0.965, *T*_max_ = 0.984
7616 measured reflections
1978 independent reflections
679 reflections with *I* > 2σ(*I*)
*R*_int_ = 0.082

#### Refinement

*R*[*F* ^2^ > 2σ(*F* ^2^)] = 0.067
*wR*(*F* ^2^) = 0.149
*S* = 1.01
1978 reflections
159 parameters
H-atom parameters constrained
Δρ_max_ = 0.14 e Å^−3^
Δρ_min_ = −0.14 e Å^−3^

### 

Data collection: *SMART* (Bruker, 2007 ▶); cell refinement: *SAINT* (Bruker, 2007 ▶); data reduction: *SAINT*; program(s) used to solve structure: *SHELXS97* (Sheldrick, 2008 ▶); program(s) used to refine structure: *SHELXL97* (Sheldrick, 2008 ▶); molecular graphics: *SHELXTL* (Sheldrick, 2008 ▶); software used to prepare material for publication: *SHELXTL*.

## Supplementary Material

Crystal structure: contains datablocks global, I. DOI: 10.1107/S1600536808043134/lh2745sup1.cif

Structure factors: contains datablocks I. DOI: 10.1107/S1600536808043134/lh2745Isup2.hkl

Additional supplementary materials:  crystallographic information; 3D view; checkCIF report

## Figures and Tables

**Table 1:** Selected torsion angles (°)

C7—C1—C2—C3	179.5 (4)
C3—C4—C5—C8	179.5 (4)
C4—C5—C6—O3	−179.5 (4)
C8—C5—C6—C1	−180.0 (4)
C7—C1—C6—C5	−179.2 (4)
C6—C1—C7—O2	174.1 (4)
N1^i^—N1—C8—C5	−179.4 (4)
C6—C5—C8—N1	−173.9 (4)
C11—N2—C9—O4	178.3 (5)

Symmetry code: (i) 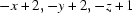.

**Table 2:** Hydrogen-bond geometry (Å, °)

*D*—H⋯*A*	*D*—H	H⋯*A*	*D*⋯*A*	*D*—H⋯*A*
C9—H9⋯O1^ii^	0.93	2.59	3.261 (6)	130
C2—H2*A*⋯O2	0.93	2.43	2.742 (6)	99
O3—H3⋯O1	0.82	1.85	2.568 (4)	146
O2—H2⋯O4^iii^	0.82	1.76	2.557 (4)	162

Symmetry codes: (ii) 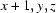; (iii) 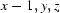.

**Fig. 1.**
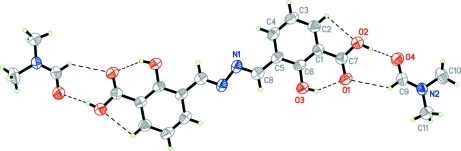
The molecular structure with displacement ellipsoids at the 30% probability level. Hydrogen bonds show as dashed lines.

## supplementary crystallographic information

### Comment

Schiff bases are one of most prevalent mixed-donor ligands in the field of coordination chemistry. Schiff bases have been used widely as ligands in the formation of transition metal complexes. There has been growing interest in Schiff base ligands, mainly because of their wide application in the field of biochemistry, synthesis, and catalysis (Pal *et al.*, 2005; Hou *et al.*, 2001; Ren *et al.*, 2002).

Herein, we report the synthesis and crystal structure of the Schiff-base compound, (I). The molecule lies across a crystallographic inversion centre which is situated at the midpoint of the N—N (1.393 (6) Å) bond. The molecular structure of (I) is shown in Fig.1.  All bond lengths are within in normal ranges (Allen *et al.*, 1987). The N1—C8 [1.281 (5) Å] and N1—N1^i^ [1.393 (6) Å] (symmetry code: (i) -x+2, -y+2, -z+1) distances indicate these correspond to double and single bonds, respectively. The torsion angles indicate that the molecule is essentially planar with the C=N bond adjacent to the benzene rings adopting a *trans* configuration with resect to its substitution. Intramolecular O—H···O and C—H···O hydrogen bonds form S(6) and S(5) ring motifs, respectively (Bernstein *et al.*, 1995). The Schiff-base molecule and solvent DMF molecules are connected by intermolecular hydrogen bonds (Fig.1 and Table. 2). Some crystal structures which are closely related to the title compound have already been studied (Chattopadhyay *et al.*,2008; Cucos *et al.*, 2006; Fu, 2007; Mijanuddina *et al.*, 2004; Dreuw *et al.*, 2005; Sreerama *et al.*, 2007).

### Experimental

Reagents and solvents used were of commercially available quality. To a stirred solution of 3-formylsalicylic acid (0.332 g, 2 mmol) in absolute methanol (10 ml) was added dropwise hydrazine hydrate (0.050 g, 1 mmol). After a few minutes, an orange precipitate appeared, which was isolated by filtration, washed with methanol, and dried in air. Crystals of (I) suitable for X-ray diffraction were obtained by recrystallized the crude product from DMF solution.

### Refinement

H atoms were positioned geometrically and refined using a riding model, with C—H = 0.93–0.96 Å and with *U*_iso_(H) = 1.2*U*_eq_(C) or 1.5*U*_eq_(C) for methyl H atoms.

### Crystal data

**Table:**

C_16_H_12_N_2_O_6_·2C_3_H_7_NO	*F*(000) = 500
*M**_r_* = 474.47	*D*_x_ = 1.311 Mg m^−^^3^
Monoclinic, *P*2_1_/*c*	Melting point: 443 K
Hall symbol: -P 2ybc	Mo *K*α radiation, λ = 0.71073 Å
*a* = 5.9136 (12) Å	Cell parameters from 812 reflections
*b* = 10.837 (2) Å	θ = 2.1–15.4°
*c* = 18.991 (4) Å	µ = 0.10 mm^−^^1^
β = 98.96 (3)°	*T* = 295 K
*V* = 1202.2 (4) Å^3^	Needle, red
*Z* = 2	0.36 × 0.20 × 0.16 mm

### Data collection

**Table:**

Bruker SMART CCD area-detector diffractometer	1978 independent reflections
Radiation source: fine-focus sealed tube	679 reflections with *I* > 2σ(*I*)
graphite	*R*_int_ = 0.082
φ and ω scans	θ_max_ = 25.0°, θ_min_ = 2.2°
Absorption correction: multi-scan (*SADABS*; Sheldrick, 1996)	*h* = −6→7
*T*_min_ = 0.965, *T*_max_ = 0.984	*k* = −12→12
7616 measured reflections	*l* = −22→22

### Refinement

**Table:**

Refinement on *F*^2^	Secondary atom site location: difference Fourier map
Least-squares matrix: full	Hydrogen site location: inferred from neighbouring sites
*R*[*F*^2^ > 2σ(*F*^2^)] = 0.067	H-atom parameters constrained
*wR*(*F*^2^) = 0.149	*w* = 1/[σ^2^(*F*_o_^2^) + (0.036*P*)^2^ + 0.022*P*] where *P* = (*F*_o_^2^ + 2*F*_c_^2^)/3
*S* = 1.01	(Δ/σ)_max_ < 0.001
1978 reflections	Δρ_max_ = 0.14 e Å^−^^3^
159 parameters	Δρ_min_ = −0.13 e Å^−^^3^
0 restraints	Extinction correction: *SHELXL*, Fc^*^=kFc[1+0.001xFc^2^λ^3^/sin(2θ)]^-1/4^
Primary atom site location: structure-invariant direct methods	Extinction coefficient: 0.016 (4)

### Special details

**Table:**

Geometry. All e.s.d.'s (except the e.s.d. in the dihedral angle between two l.s. planes) are estimated using the full covariance matrix. The cell e.s.d.'s are taken into account individually in the estimation of e.s.d.'s in distances, angles and torsion angles; correlations between e.s.d.'s in cell parameters are only used when they are defined by crystal symmetry. An approximate (isotropic) treatment of cell e.s.d.'s is used for estimating e.s.d.'s involving l.s. planes.
Refinement. Refinement of *F*^2^ against ALL reflections. The weighted *R*-factor *wR* and goodness of fit *S* are based on *F*^2^, conventional *R*-factors *R* are based on *F*, with *F* set to zero for negative *F*^2^. The threshold expression of *F*^2^ > σ(*F*^2^) is used only for calculating *R*-factors(gt) *etc*. and is not relevant to the choice of reflections for refinement. *R*-factors based on *F*^2^ are statistically about twice as large as those based on *F*, and *R*- factors based on ALL data will be even larger.

### Fractional atomic coordinates and isotropic or equivalent isotropic displacement parameters (Å^2^)

**Table:**

	*x*	*y*	*z*	*U*_iso_*/*U*_eq_	
N1	0.9469 (6)	0.9471 (3)	0.51035 (19)	0.0850 (12)	
N2	0.2954 (7)	0.6509 (3)	0.23059 (19)	0.0830 (11)	
O1	−0.0194 (5)	0.7647 (3)	0.34383 (15)	0.0925 (10)	
O2	−0.0718 (5)	0.5919 (3)	0.40230 (16)	0.0978 (10)	
H2	−0.1983	0.5948	0.3775	0.147*	
O3	0.3501 (5)	0.8896 (3)	0.37878 (14)	0.0913 (10)	
H3	0.2293	0.8697	0.3541	0.137*	
O4	0.5660 (5)	0.5621 (3)	0.31107 (17)	0.1024 (11)	
C1	0.2520 (8)	0.7119 (4)	0.4437 (2)	0.0735 (12)	
C2	0.3105 (8)	0.6335 (4)	0.5012 (2)	0.0901 (14)	
H2A	0.2151	0.5678	0.5079	0.108*	
C3	0.5105 (10)	0.6525 (5)	0.5488 (2)	0.1046 (16)	
H3A	0.5491	0.6001	0.5877	0.126*	
C4	0.6512 (8)	0.7496 (5)	0.5380 (2)	0.0977 (16)	
H4	0.7858	0.7614	0.5698	0.117*	
C5	0.5984 (8)	0.8308 (4)	0.4808 (2)	0.0753 (13)	
C6	0.3970 (8)	0.8102 (4)	0.4342 (2)	0.0717 (12)	
C7	0.0427 (8)	0.6924 (5)	0.3928 (3)	0.0782 (13)	
C8	0.7514 (9)	0.9333 (4)	0.4714 (2)	0.0829 (13)	
H8	0.7050	0.9909	0.4357	0.099*	
C9	0.4849 (9)	0.6507 (5)	0.2750 (3)	0.0863 (14)	
H9	0.5674	0.7240	0.2801	0.104*	
C10	0.1512 (9)	0.5403 (4)	0.2220 (2)	0.1212 (18)	
H10A	0.2158	0.4811	0.1933	0.182*	
H10B	0.0001	0.5622	0.1993	0.182*	
H10C	0.1433	0.5052	0.2680	0.182*	
C11	0.2066 (9)	0.7591 (5)	0.1912 (3)	0.1217 (18)	
H11A	0.3080	0.8274	0.2039	0.183*	
H11B	0.0579	0.7784	0.2024	0.183*	
H11C	0.1949	0.7432	0.1410	0.183*	

### Atomic displacement parameters (Å^2^)

**Table:**

	*U*^11^	*U*^22^	*U*^33^	*U*^12^	*U*^13^	*U*^23^
N1	0.066 (3)	0.093 (3)	0.091 (3)	0.006 (2)	0.000 (2)	−0.007 (2)
N2	0.079 (3)	0.082 (3)	0.085 (3)	−0.017 (2)	0.005 (2)	−0.005 (2)
O1	0.093 (2)	0.093 (2)	0.084 (2)	0.0066 (18)	−0.0070 (18)	0.0125 (17)
O2	0.089 (2)	0.093 (2)	0.107 (3)	−0.003 (2)	0.0012 (19)	0.0162 (19)
O3	0.096 (2)	0.094 (2)	0.079 (2)	−0.0056 (18)	−0.0022 (17)	0.0103 (17)
O4	0.098 (3)	0.085 (2)	0.116 (3)	−0.001 (2)	−0.010 (2)	0.0036 (19)
C1	0.071 (3)	0.078 (3)	0.072 (3)	0.009 (3)	0.014 (3)	0.000 (3)
C2	0.092 (4)	0.098 (3)	0.080 (3)	0.001 (3)	0.015 (3)	0.008 (3)
C3	0.104 (4)	0.124 (4)	0.080 (3)	0.018 (4)	−0.003 (3)	0.022 (3)
C4	0.085 (4)	0.119 (4)	0.082 (4)	0.009 (3)	−0.009 (3)	0.005 (3)
C5	0.083 (3)	0.077 (3)	0.063 (3)	0.021 (3)	0.002 (3)	−0.008 (3)
C6	0.078 (3)	0.075 (3)	0.062 (3)	0.021 (3)	0.011 (3)	0.001 (2)
C7	0.075 (3)	0.069 (3)	0.090 (4)	0.013 (3)	0.012 (3)	−0.006 (3)
C8	0.089 (4)	0.079 (3)	0.082 (3)	0.020 (3)	0.018 (3)	−0.009 (2)
C9	0.076 (4)	0.090 (4)	0.094 (4)	−0.012 (3)	0.014 (3)	−0.007 (3)
C10	0.126 (5)	0.116 (4)	0.123 (4)	−0.042 (4)	0.023 (4)	−0.037 (3)
C11	0.118 (4)	0.118 (4)	0.120 (4)	−0.009 (3)	−0.008 (3)	0.023 (3)

### Geometric parameters (Å, °)

**Table:**

N1—C8	1.281 (5)	C2—H2A	0.9300
N1—N1^i^	1.393 (6)	C3—C4	1.377 (6)
N2—C9	1.293 (5)	C3—H3A	0.9300
N2—C11	1.445 (5)	C4—C5	1.395 (5)
N2—C10	1.465 (5)	C4—H4	0.9300
O1—C7	1.228 (5)	C5—C6	1.387 (5)
O2—C7	1.310 (5)	C5—C8	1.462 (6)
O2—H2	0.8200	C8—H8	0.9300
O3—C6	1.354 (4)	C9—H9	0.9300
O3—H3	0.8200	C10—H10A	0.9600
O4—C9	1.232 (5)	C10—H10B	0.9600
C1—C2	1.384 (5)	C10—H10C	0.9600
C1—C6	1.397 (5)	C11—H11A	0.9600
C1—C7	1.462 (5)	C11—H11B	0.9600
C2—C3	1.388 (6)	C11—H11C	0.9600
			
C8—N1—N1^i^	109.8 (5)	C5—C6—C1	121.6 (4)
C9—N2—C11	123.2 (4)	O1—C7—O2	122.3 (4)
C9—N2—C10	120.1 (4)	O1—C7—C1	122.1 (5)
C11—N2—C10	116.6 (4)	O2—C7—C1	115.6 (4)
C7—O2—H2	109.5	N1—C8—C5	122.5 (4)
C6—O3—H3	109.5	N1—C8—H8	118.7
C2—C1—C6	119.1 (4)	C5—C8—H8	118.7
C2—C1—C7	121.0 (5)	O4—C9—N2	125.9 (5)
C6—C1—C7	119.9 (4)	O4—C9—H9	117.0
C1—C2—C3	120.4 (5)	N2—C9—H9	117.0
C1—C2—H2A	119.8	N2—C10—H10A	109.5
C3—C2—H2A	119.8	N2—C10—H10B	109.5
C4—C3—C2	119.3 (4)	H10A—C10—H10B	109.5
C4—C3—H3A	120.4	N2—C10—H10C	109.5
C2—C3—H3A	120.4	H10A—C10—H10C	109.5
C3—C4—C5	122.1 (5)	H10B—C10—H10C	109.5
C3—C4—H4	118.9	N2—C11—H11A	109.5
C5—C4—H4	118.9	N2—C11—H11B	109.5
C6—C5—C4	117.5 (5)	H11A—C11—H11B	109.5
C6—C5—C8	122.0 (4)	N2—C11—H11C	109.5
C4—C5—C8	120.5 (4)	H11A—C11—H11C	109.5
O3—C6—C5	116.5 (5)	H11B—C11—H11C	109.5
O3—C6—C1	121.9 (4)		
			
C6—C1—C2—C3	0.1 (6)	C2—C1—C6—C5	0.3 (6)
C7—C1—C2—C3	179.5 (4)	C7—C1—C6—C5	−179.2 (4)
C1—C2—C3—C4	−0.5 (7)	C2—C1—C7—O1	175.7 (4)
C2—C3—C4—C5	0.6 (8)	C6—C1—C7—O1	−4.9 (6)
C3—C4—C5—C6	−0.3 (7)	C2—C1—C7—O2	−5.4 (6)
C3—C4—C5—C8	179.5 (4)	C6—C1—C7—O2	174.1 (4)
C4—C5—C6—O3	−179.5 (4)	N1^i^—N1—C8—C5	−179.4 (4)
C8—C5—C6—O3	0.7 (6)	C6—C5—C8—N1	−173.9 (4)
C4—C5—C6—C1	−0.2 (6)	C4—C5—C8—N1	6.4 (6)
C8—C5—C6—C1	−180.0 (4)	C11—N2—C9—O4	178.3 (5)
C2—C1—C6—O3	179.5 (4)	C10—N2—C9—O4	2.7 (7)
C7—C1—C6—O3	0.1 (6)		

Symmetry codes: (i) −*x*+2, −*y*+2, −*z*+1.

### Hydrogen-bond geometry (Å, °)

**Table:**

*D*—H···*A*	*D*—H	H···*A*	*D*···*A*	*D*—H···*A*
C9—H9···O1^ii^	0.93	2.59	3.261 (6)	130
C2—H2A···O2	0.93	2.43	2.742 (6)	99
O3—H3···O1	0.82	1.85	2.568 (4)	146
O2—H2···O4^iii^	0.82	1.76	2.557 (4)	162

Symmetry codes: (ii) *x*+1, *y*, *z*; (iii) *x*−1, *y*, *z*.

## References

[bb1] Allen, F. H., Kennard, O., Watson, D. G., Brammer, L., Orpen, A. G. & Taylor, R. (1987). *J. Chem. Soc. Perkin Trans. 2*, pp. S1–19.

[bb2] Bernstein, J., Davis, R. E., Shimoni, L. & Chang, N. L. (1995). *Angew. Chem. Int. Ed. Engl.* **34**, 1555–1573.

[bb3] Bruker (2007). *SMART* and *SAINT* Bruker AXS Inc., Madison, Wisconsin, USA.

[bb4] Butcher, R. J., Bendre, R. S. & Kuwar, A. S. (2007). *Acta Cryst.* E**63**, o3360.

[bb5] Chattopadhyay, B., Basu, S., Ghosh, S., Helliwell, M. & Mukherjee, M. (2008). *Acta Cryst.* E**64**, o866. 10.1107/S1600536808010155 PMC2961147 21202353

[bb6] Cucos, P., Pascu, M., Sessoli, R., Avarvari, N., Pointillart, F. & Andruh, M. (2006). *Inorg. Chem.* **45**, 7035–7037. 10.1021/ic060533u 16933896

[bb7] Dreuw, A., Plötner, J., Lorenz, L., Wachtveitl, J., Djanhan, J. E., Brüning, J., Metz, T., Bolte, M. & Schmidt, M. U. (2005). *Angew. Chem. Int. Ed.* **44**, 7783–7786. 10.1002/anie.200501781 16315162

[bb8] Fu, Z.-W. (2007). *Acta Cryst.* E**63**, o2993.

[bb9] Hou, B., Friedman, N., Ruhman, S., Sheves, M. & Ottolenghi, M. J. (2001). *Phys. Chem. B.* **105**, 7042–7048.

[bb10] Mijanuddin, M., Sheldrick, W. S., Mayer-Figge, H., Ali, M. & Chattopadhyay, N. (2004). *J. Mol. Struct.*, **693**, 161–165.

[bb11] Pal, S., Barik, A. K., Gupta, S., Hazra, A., Kar, S. K., Peng, S.-M., Lee, G.-H., Butcher, R. J., El Fallah, M. S. & Ribas, J. (2005). *Inorg. Chem.* **44**, 3880–3889. 10.1021/ic0501420 15907114

[bb12] Ren, S., Wang, R., Komatsu, K., Bonaz-Krause, P., Zyrianov, Y., McKenna, C. E., Csipke, C., Tokes, Z. A. & Lien, E. J. (2002). *J. Med. Chem.* **45**, 410–419. 10.1021/jm010252q 11784145

[bb13] Sheldrick, G. M. (1996). *SADABS* University of Göttingen, Germany.

[bb14] Sheldrick, G. M. (2008). *Acta Cryst.* A**64**, 112–122. 10.1107/S0108767307043930 18156677

[bb15] Sreerama, S. G., Mukhopadhyay, A. & Pal, S. (2007). *Polyhedron*, **26**, 4101–4106.

